# Psychiatric outcomes after primary and secondary earthquake exposure: effects of loss, media, and treatment

**DOI:** 10.3389/fpsyt.2025.1715590

**Published:** 2026-01-27

**Authors:** Hüseyin Ozan Torun, Burak Akdöner, Talat Sarıkavak, Umut Kirli

**Affiliations:** 1 Department of Psychiatry Izmir City Hospital, Izmir, Türkiye; 2 Ege University Institute on Drug Abuse, Toxicology and Pharmaceutical Science, Izmir, Türkiye; 3 Department of Psychiatry, Near East University Faculty of Medicine, Nicosia, Cyprus; 4 Department of Psychiatry, Istanbul Atlas University Faculty of Medicine, Istanbul, Türkiye

**Keywords:** early intervention, earthquake, media exposure, post-traumatic stress, therapy

## Abstract

**Introduction:**

Earthquakes can cause substantial psychiatric morbidity including Post-Traumatic Stress Disorder (PTSD). While direct exposure effects are well known less is understood about relapse after indirect exposure. Following the 2023 Kahramanmaraş earthquakes clinicians observed renewed distress among survivors of the 2020 Aegean Sea earthquake. Understanding how media exposure individual factors contribute to symptom reactivation is important for post-disaster care. This study investigated factors linked to PTSD symptom relapse after repeated disaster exposure.

**Method:**

This prospective quantitative study (February 2023–February 2024) recruited patients from a psychiatry outpatient clinic following the Kahramanmaraş earthquakes. Individuals with earthquake-related complaints who had also experienced the 2020 Aegean Sea earthquake were enrolled. Post-traumatic stress symptoms were assessed using the Post-Traumatic Stress Disorder Checklist for DSM-5 (PCL-5) at baseline and at 1, 3, 6, 9, and 12 months. Resilience was measured using the Resilience Scale for Adults (RSA) at baseline. Sociodemographic and clinical variables, including prior psychiatric treatment and media exposure type, were recorded. Multivariate analysis of variance was used to examine longitudinal symptom trajectories and predictors.

**Results:**

The study included 87 participants (mean age = 48.8 ± 12.5yrs), the majority were female (85%). Following the Aegean Sea earthquake, 45% of participants received psychiatric treatment, with loss experience being the only significant predictor of treatment seeking (χ² = 14.90, p <.001). After the Kahramanmaraş earthquakes, 74.7% received psychiatric treatment. A significant effect of time on PCL-5 symptom severity across the 12-month follow-up (Pillais Trace = .40, F = 7.32, p <.001). Age and resilience were independently associated with PTSD severity, with resilience showing the strongest effect (F = 40.24, p <.001). Higher PCL-5 scores among those primarily exposed to traditional media (F = 5.11, p = .027), symptom trajectories did not differ over time.

**Conclusion:**

This study shows that indirect exposure to large-scale disasters can shape symptom trajectories in previously affected individuals. Resilience served as a protective factor, while prior treatment indicated both vulnerability and adaptive potential. Media exposure type also influenced symptom severity. These results highlight the value of assessing resilience, treatment history, and media use in trauma-informed care after repeated disaster exposure.

## 1 Introduction

Earthquakes have many different devastating consequences, including the disruptive effects on mental health. Post-earthquake acute stress reactions, sleep problems, fear and anxiety, and more serious psychiatric conditions such as post-traumatic stress disorder (PTSD) can be some of these psychiatric consequences (1).

Post-Traumatic Stress Disorder (PTSD) symptoms can vary significantly in clinical practice but in general there are four symptom dimensions, these are; intrusive thoughts, avoidance behaviour, negative changes in mood and cognition, and hyperarousal symptoms. Individuals may experience flashbacks, nightmares, or distressing memories of the traumatic event. Avoidance from reminders of the trauma, including people, places, or activities are common in PTSD. It is a quite disruptive disorder for people suffering from it and cause significant dysfunctions (2). According to DSM-5 TR criteria, post-traumatic stress disorder can be triggered through exposure to a traumatic event, direct experience, or witnessing real or threatening events involving death, serious injury, or sexual violence (3).

The 2020 Aegean Sea earthquake occurred on October 30, 2020, 23 km from the Seferihisar district of Izmir, with a magnitude of 6.9. In Türkiye and Greece, 119 people died and 1053 people were injured. After this earthquake, many people were observed to have symptoms of depression, anxiety and post-traumatic stress disorder and received treatment (4). Experiencing an earthquake causes similar psychiatric symptoms all around the world. A review study reported that, on average, 24% of individuals affected by disasters develop post-traumatic stress disorder (PTSD), 28% experience depressive symptoms, and 23% develop an anxiety disorder within the first six months following the event (5). Another meta-analysis of the post-earthquake period following the 2010 Haiti earthquake found that approximately one in four individuals experienced severe symptoms of PTSD, one in three reported severe depressive symptoms, and one in five experienced severe anxiety symptoms in the aftermath of the disaster (6).

While this is the case for those who experience an earthquake first-hand, the occurrence of a similar incident nearby can trigger a relapse in psychiatric conditions. Twenty-seven months later from the Aegean Sea earthquake, two major earthquakes centered in Kahramanmaraş on February 6, 2023 caused great destruction in Türkiye. The city of Kahramanmaraş experienced an earthquake measuring 7.7 on the Richter scale, and a second earthquake measuring 7.6 occurred in the same province approximately 10 hours after the earthquake on the same day. These two earthquakes affected a large area covering 11 cities in Türkiye. Total population of these 11 cities constitute approximately 15% of the population of Türkiye. According to official reports, these earthquakes caused more than 50, 000 deaths and 100, 000 injuries (7). Although several mechanisms may account for this apparent symptom relapse, such as cumulative stress, unresolved trauma, socioeconomic disruption, and displacement, post-earthquake media exposure appears to be a potentially influential factor. A growing body of evidence suggests that sustained exposure to disaster-related media content may heighten trauma responses, particularly among previously exposed or vulnerable populations (8). However, the *type* of media consumed may play a fundamental role in shaping the intensity and persistence of emotional responses.

Traditional media sources (e.g., television broadcasts, radio, newspapers) typically deliver structured, temporally bounded, and editorially curated information. Under certain conditions, such coverage may foster a sense of social cohesion, collective mourning, or shared understanding of the event, thereby potentially supporting resilience (9). Traditional outlets may also filter excessively graphic content, maintain narrative coherence, and regulate the pace of information flow.

In contrast, social media platforms provide a highly dynamic, unfiltered, and algorithmically driven information environment, where emotionally salient or sensational content is prioritized by engagement-based algorithms. This creates continuous exposure to graphic images, personal testimonies, and distressing footage, often without contextual framing. The rapid dissemination and repetitive availability of such content amplify the risk of emotional contagion, where negative emotional states spread within online networks (10). Furthermore, repeated viewing of traumatic imagery, even indirectly or at a distance, may induce vicarious traumatization, producing symptoms similar to those associated with direct trauma exposure (1). Thus, differentiating between traditional and social media is theoretically meaningful and clinically relevant. However, most existing studies treat media exposure as a single, undifferentiated construct, leaving a notable gap regarding the distinct psychological impacts of these two media types.

One other contributing area may be individuals prior trauma histories, psychosocial resources, and pre-existing vulnerabilities (11, 12). Understanding these determinants becomes even more critical in contexts of repeated disaster exposure, where individuals encounter secondary traumatic events before fully recovering from the initial one. One factor that has received relatively little empirical attention is the role of previous engagement with mental health services following an initial disaster. On one hand, theoretical models of emotional reactivity suggest that earlier trauma can prime neurobiological systems to respond more intensely to later stressors, a phenomenon often referred to as stress sensitization (13). Individuals with a history of psychiatric symptoms or treatment might therefore enter subsequent disaster contexts with a more activated threat-detection system. On the other hand, engagement with mental health treatment can promote the development of protective psychological capacities. Exposure-based therapies, cognitive restructuring, and supportive clinical relationships are known to strengthen adaptive coping, improve emotional regulation, and increase readiness to seek help during future distress (14). These gains are often reflected in higher resilience indicators or more rapid clinical stabilization when new stressors arise. Thus, prior treatment may represent a complex marker that incorporates both vulnerability and adaptive potential.

Despite conceptual frameworks acknowledging this duality, very few empirical studies have examined how prior treatment following an initial disaster influences psychological functioning during subsequent disaster exposure within the same population. Even fewer have incorporated resilience and treatment characteristics to elucidate how vulnerability and adaptive capacities interact. Filling this gap is essential for optimizing early intervention strategies and for identifying which individuals may require enhanced monitoring during future disasters.

Against this background, the primary aim of the present study was to investigate the factors associated with the relapse of psychiatric symptoms among individuals who experienced the 2020 Aegean Sea earthquake and were subsequently exposed to the 2023 Kahramanmaraş earthquakes. Specifically, we aimed to examine whether the type of media exposure (traditional vs. social media) contributes differently to the severity of post-disaster symptom relapse, given the distinct emotional, cognitive, and behavioral mechanisms associated with each medium. A second aim was to identify and clinical variables that may help explain variations in relapse severity. Identifying such factors may contribute to early risk stratification and guide targeted public mental health interventions in post-disaster contexts. Finally, the study assessed an *a priori* hypothesis: individuals who received psychiatric support following the 2020 earthquake were expected to report milder symptoms after the 2023 earthquakes compared to those who did not receive prior support. This hypothesis is grounded in evidence suggesting that early therapeutic intervention fosters adaptive coping strategies, enhances emotional regulation, and strengthens resilience, which may collectively reduce vulnerability to symptom recurrence when encountering subsequent traumatic events.

## 2 Materials and methods

Our study was designed as quantitative, analytical and prospective. Data were collected between February 2023 - February 2024. The study utilized a consecutive, non-probability sampling technique. Participants were identified and recruited exclusively within the Psychiatry Outpatient Clinic of Seferihisar Necat Hepkon State Hospital in Izmir. The selection process was based on screening for earthquake-related complaints: following the 2023 Kahramanmaraş earthquakes, all individuals presenting to the clinic with new or exacerbated psychological symptoms (anxiety, insomnia, re-experiencing symptoms) were consecutively screened. Those who confirmed having experienced the 2020 Aegean Sea earthquake and met all other inclusion/exclusion criteria were invited to participate. The final sample of 87 individuals was therefore voluntary, consisting of individuals who actively sought clinical help for their symptoms and provided written informed consent. This method ensures the sample is representative of the help-seeking population experiencing symptom relapse, rather than the general population of 2020 earthquake survivors.

All participants gave written informed consent before the enrollment. Participants were included in the study after an evaluation of the inclusion and exclusion criteria.

Participants were included in the study if they were 18 years of age or older, had experienced the 2020 Aegean Sea earthquake in Seferihisar. Participants were excluded if they had a diagnosed mental disorder or intellectual disability that could impair the reliability of questionnaire responses. Individuals were also excluded if they were present in the 2023 Kahramanmaraş earthquake zone at any time during the research process (relief work, assignment, transfer, migration, etc.), experienced the loss of a close friend or relative due to the 2023 Kahramanmaraş earthquake, or obtained direct information from the earthquake region outside of media channels. Failure to attend scheduled outpatient clinic examinations or declining participation after being informed also resulted in exclusion.

The Resilience Scale for Adults (RSA) was developed to assess protective factors that support resilience in adults (15). The validity and reliability of the Turkish version were established by Basım and Çetin (16).

The Post-Traumatic Stress Disorder Checklist for DSM-5 (PCL-5) is a 20-item self-report measure designed to assess PTSD symptoms according to DSM-5 criteria. It can be used for screening, diagnostic assessment, and monitoring symptom changes over time (17). The Turkish version of the PCL-5 was validated by Boysan et al. (18). Strong correlations between PCL-5 scores and other trauma-related symptom measures supported its construct validity. A cutoff score of ≥47 was recommended for the diagnosis of PTSD, with a sensitivity of 0.76 and a specificity of 0.69. In this study, the 47 cutoff score was used solely for descriptive purposes to illustrate the severity of symptoms at the baseline visit. For all subsequent longitudinal and comparative analyses, the PCL-5 was utilized exclusively as a continuous variable to measure the severity and trajectory of post-traumatic stress symptoms over time.

After the Kahramanmaraş earthquake, individuals presenting to the outpatient clinic with earthquake-related psychological complaints were screened for eligibility. During this initial assessment, patients were first asked about their experience of the 2020 Aegean Sea earthquake, and inclusion and exclusion criteria were applied. Participants were also asked whether they had received any psychological intervention (psychotherapy and/or pharmacological treatment) following the 2020 event, as this variable constituted part of the studys hypothesis. In addition, sociodemographic information (age, sex, marital status, occupational status etc.) was collected.

### 2.1 Baseline assessment

At the first clinic assessment, two standardized measures were administered: the Resilience Scale for Adults (RSA) and the Post-Traumatic Stress Disorder Checklist for DSM-5 (PCL-5). The RSA was used to evaluate protective factors associated with resilience, allowing us to determine whether baseline resilience moderated symptom relapse after the second earthquake. The PCL-5 was administered to assess current PTSD symptom severity. In addition, participants were asked how they followed news about the Kahramanmaraş earthquake (e.g., television, newspapers, radio, or social media), enabling comparison between different types of media exposure. Information regarding previous or ongoing treatments (type, duration, and modality) was also recorded.

### 2.2 Follow-up assessments

To monitor changes in symptom severity over time, the PCL-5 was re-administered at 1, 3, 6, 9, and 12 months after the baseline visit. Repeated administration of the PCL-5 allowed the study to track symptom trajectories and identify cases of relapse or sustained improvement.

To ensure high data quality and consistency throughout the 12-month follow-up period, all assessments (PCL-5 and RSA administration) were conducted by the same psychiatrist. Assessment procedures were highly standardized across all time points (baseline, 1, 3, 6, 9, and 12 months) to minimize potential interviewer or administrator bias. This standardization included consistent questionnaire completion instructions and the same follow-up schedules for all participants.

In the analysis conducted to determine the sample size, it was determined that in order to obtain an effect size of f=0.3, a type 1 error margin of 0.05, and a power of 0.80, a total of 80 people were required. Considering the reserve participants, a total of 90 people were planned to be included in the study.

A total of 237 individuals who presented to the Psychiatry Outpatient Clinic with earthquake-related psychological complaints following the 2023 Kahramanmaraş earthquakes were initially screened for participation. Of these, 143 individuals were excluded, primarily for the following reasons: Did not experience the 2020 Aegean Sea earthquake (*n* = 52), Lost a close friend or relative in the 2023 Kahramanmaraş earthquake (*n* = 22), Had information about the earthquake zone outside of media exposure (*n* = 48), Refused to participate in the study (*n* = 2), Met other exclusion criteria (*n* = 19). Consequently, 95 participants met all eligibility criteria and provided informed consent. During the 12-month prospective follow-up period, a total of 8 participants (8.42**%**) dropped out from the study,.The final analysis included data from 87 participants who completed the full 12-month follow-up protocol.

Analyses were structured to examine cross-sectional associations between media exposure, prior treatment, and symptom severity at baseline, and longitudinal changes in PCL-5 scores over 12 months, while adjusting for key sociodemographic covariates. Statistical analyses were conducted using SPSS 25. Normality was assessed with the Shapiro–Wilk test. Normally distributed variables were presented as mean and standard deviations; non-normal variables as median with minimum and maximum values. Group comparisons used the Independent t-test for parametric data, the Mann–Whitney U test for non-parametric data, and the Chi-Square or Fishers Exact test for categorical variables.Age, gender, marital status, educational status, employment status, media exposure, experiencing loss in the Aegean Sea Earthquake, type of prior loss, were used as the independent variables. The Resilience Scale for Adults (RSA) scores, PCL-5 scores, psychiatric treatment after Kahramanmaraş Earthquake was used as the dependent variable. Psychiatric treatment after Aegean Sea Earthquake was used both as dependent and independent variables. Logistic regression analyses were performed to determine the predictive values of significant variables. A repeated-measures multivariate analysis of variance (RM-MANOVA) was conducted to reduce Type I error. These sociodemographic variables were included as covariates in regression and RM-MANOVA models. Assumptions of sphericity and covariance equality were evaluated using Mauchlys and Boxs M tests, violations were addressed using Greenhouse–Geisser corrections and Pillais Trace. Polynomial contrasts were used to examine the functional form of temporal change. Bonferroni-adjusted pairwise comparisons based on estimated marginal means were applied for *post hoc* testing. Between-subjects main effects and time × factor interactions were evaluated via multivariate and univariate tests. Effect sizes (partial η²) and observed power were reported. The level of significance was set at p<0.05. Following the reporting of the dropout rate during the 12-month follow-up, missing data for the PCL-5 and RSA follow-up scores due to attrition were handled using the Listwise Deletion method for all comparative and regression analyses. Specifically, only participants with complete data for the variables used in a given analysis were included in that specific test.

## 3 Results

Our sample consisted of 87 participants (M age = 48.79 years, SD = 12.47, range: 23-72). 52.9% (n=46) were employed. Mean education duration was 10.94 years (± 2.635, min:5-max:17). Gender distribution was 85% female (n=74) and 15% male (n=13). Regarding marital status 72, 4% (n=63) of the participants were married, 27, 6% (n=24) were single. Detailed data regarding sample characteristics are summarized in **Table 1**.

**Table 1:** Sample characteristics.

Total sample (n): 87		Variables	n (%)
Experiencing Loss in the Aegean Sea Earthquake	Yes	Human Loss	15 (17.2)
		Material Loss	48 (55.1)
	No		24 (27.6)
Psychiatric treatment after Aegean Sea Earthquake	Yes	Pharmacotherapy	18 (20.7)
		Psychotherapy	4(4.6)
		Both	18(20.7)
	No		47(54)
Media exposure after Kahramanmaraş earthquake		Traditional media	39(44.8)
		Social media	48(55.2)
Psychiatric treatment after Kahramanmaraş Earthquake		Yes	65(74.7
		No	22(25.3)

### 3.1 Aegean Sea earthquake

No significant differences were found between sociodemographic variable, such as gender, age, marital status, or occupational status in relation to seeking psychiatric treatment. The only significant factor was experiencing loss; as expected, participants who had suffered loss were significantly more likely to seek psychiatric support (χ² = 14.95, *p* < 0.001). Detailed data regarding sample characteristics are summarized in **Table 2**.

**Table 2:** Comparison of clinical characteristics between the participants who had psychiatric support and those who did not.

Psychiatric treatment		Yes (n=40)	No (n=47)	Statistics
Gender	Male	7	6	*p=0.562*
	Female	33	41	
Living Alone	Yes	8	9	*p=0.566*
	No	32	38	
Employment	Yes	27	17	*p=0.05* *χ²=8.485*
	No	13	30	
Experiencing Loss	Yes	37	26	***p<0.001*** ***χ²=14.854***
	No	3	21	
Type of Prior Loss	Human Loss	12	3	*χ²=3.675* *p=0.055*
	Material Loss	25	23	

Bold values indicate variables with statistically significant between-group differences, with loss-related variables differing between participants who received psychiatric treatment and those who did not (p < 0.05).

A total of 40 participants (45%) received psychiatric treatment. Among them, 36 participants were administered pharmacotherapy, and of these, 18 received both pharmacotherapy and psychotherapy.

### 3.2 Kahramanmaraş earthquake

A total of 65 participants (74.7%) received psychiatric treatment after the Kahramanmaraş earthquake. Among them, 40 participants (61.5%) were prescribed selective serotonin reuptake inhibitors (SSRIs), while 25 participants (38.5%) received symptomatic pharmacological treatment. Among the participants who were required to use SSRIs, the scale scores in all controls were in favor of more severe PTSD than those who used only symptomatic treatment (**Table 3**).

**Table 3:** Comparison of scale scores among those who used SSRI or symptomatic treatment after the Kahramanmaraş earthquake.

Variables	Type of treatment	n	Scores	Statistics
The Resilience Scale for Adults (RSA)¹	Ssri	40	61.25 ± 8.918	p<0.01 *t=-11.072*
	Symptomatic	25	87.04 ± 9.481	
Treatment period (month)¹	Ssri	40	6.83 ± 2.259	p<0.01 *t=13.449*
	Symptomatic	25	1.12 ± .447	
PCL-5 first examination¹	Ssri	40	65.80 ± 4.901	p<0.01 *t=21.491*
	Symptomatic	25	40.08 ± 4.308	
PCL-5 1st month¹	Ssri	40	44.15 ± 7.510	p<0.01 *t=15.646*
	Symptomatic	25	21.68 ± 4.024	
PCL-5 3rd month¹	Ssri	40	35.13 ± 7.162	p<0.01 *t=18.777*
	Symptomatic	25	10.28 ± 3.422	
PCL-5 6th month²	Ssri	40	21 (8-44)	p<0.01 *z=-6.733*
	Symptomatic	25	1 (0-9)	
PCL-5 9th month²	Ssri	40	8.50 (0-37)	p<0.01 *z=-6.736*
	Symptomatic	25	0.00 (0-1)	
PCL-5 12th month²	Ssri	40	1.00 (0-24)	p<0.01 *z=-3.552*
	Symptomatic	25	0.00 (0-1)	

¹Independent samples t-test, mean, standart deviation, p-values and t values are given. ²Mann-Whitney U test, median, minimum-maximum, p-values and z-values are given.

Consistent with the findings from the Aegean Sea earthquake, no significant differences were found in sociodemographic characteristics between participants who received psychiatric treatment after the Kahramanmaraş earthquake and those who did not.

The repeated-measures MANOVA revealed a strong multivariate effect of time on PCL-5 scores (Pillais Trace = .40, F(5, 56) = 7.32, p <.001, η² = .40, power = .998.).

At baseline, 42 participants scored above the >47 threshold for probable PTSD. This number decreased to 14 at the 1-month assessment, 4 at the 3-month assessment, and 1 at the 6-month assessment. No participants exceeded the threshold at the 9- or 12-month follow-ups.

PCL-5 scores changed substantially across the six repeated assessments (0, 1, 3, 6, 9, and 12 months). Overall PCL-5 symptom levels varied meaningfully according to participants demographic and clinical profiles. Age was independently associated with PCL-5 scores (F = 5.94, p = .018, η² = .090), while Adult Resilience Score demonstrated a particularly strong association with outcomes (F = 40.24, p <.001, η² = .401). Years of education were not significantly related to symptom burden after adjustment. All group comparisons were estimated at the mean values of these covariates. Differences were also evident across gender, marital status, employment status, educational attainment, type of prior loss, and patterns of media exposure, indicating that both social and clinical vulnerability factors contributed to variability in symptom burden (Pillais Trace = .94, F(5, 56) = 183.65, p <.001, η² = .94, power = 1.00). A significant main effect of media exposure type was observed, F = 5.11, p = .027, η² = .078, indicating that individuals who primarily consumed traditional media had higher PCL-5 scores than those using social media. The trajectory of change was parallel across media groups, while traditional media exposure was associated with higher overall symptom levels, it did not alter the pattern or rate of symptom change. Data is provided in **Table 4**. However, there was no significant relationship between media type (traditional or social) and whether participants sought psychiatric treatment or not (χ² = 0.319, p = .375).

**Table 4:** Longitudinal changes in PCL-5 symptom severity following secondary earthquake exposure by clinical and sociodemographic factors.

Variables	Subgroups	0 months PCL-5 Mean (SE)	12 months PCL-5 Mean (SE)	95% CI (0 months)	95% CI (12 months)	Between-Subjects F	p	η²	Time × Group F (GG)	p	η²
Gender	Female	43.085 (2.648)	3.266 (1.043)	37.812–48.359	1.190–5.343	2.460	.122	.039	0.556	.604	.007
	Male	42.592 (3.847)	2.078 (1.515)	34.932–50.251	−0.938–5.094						
Marital status	Single	46.617 (3.355)	4.153 (1.321)	39.937–53.297	1.522–6.783	2.078	.155	.033	1.213	.303	.016
	Married	39.060 (3.121)	1.192 (1.229)	32.845–45.276	−1.256–3.639						
Employment	No	42.322 (5.010)	3.564 (1.973)	32.346–52.299	−0.364–7.493	1.102	.298	.018	0.857	.442	.011
	Yes	43.355 (1.999)	1.780 (0.787)	39.375–47.334	0.213–3.347						
Loss history	No	41.124 (3.584)	1.797 (1.411)	33.987–48.262	−1.014–4.607	13.411	.001	.183	1.008	.377	.013
	Yes	44.553 (2.803)	3.547 (1.104)	38.971–50.135	1.349–5.745						
Prior treatment	No	32.814 (3.645)	2.408 (1.435)	25.556–40.072	−0.450–5.266	10.666	.002	.151	27.045	.000	.260
	Yes	52.863 (2.969)	2.937 (1.169)	46.951–58.775	0.609–5.264						
Media exposure	Traditional	42.763 (3.060)	2.874 (1.205)	36.669–48.856	0.474–5.273	5.108	.027	.078	1.826	.156	.023
	Social	42.914 (3.284)	2.471 (1.293)	36.375–49.453	−0.104–5.046						

Values are adjusted estimated marginal means (SE) derived from repeated-measures GLM controlling for age, years of education, and resilience. Greenhouse–Geisser correction was applied to time × group effects.

Among participants who experienced loss during the Aegean Sea earthquake, those who had previously received psychiatric support required a significantly shorter duration of treatment following the Kahramanmaraş earthquake (M = 2.46 (months), SD = 3.12 vs. M = 4.73 (months), SD = 3.84; *p* = .012).No significant correlations were found between overall media exposure duration and either symptom severity scores or treatment duration. However, among participants who experienced loss during the Aegean Sea earthquake, low positive correlations were observed between exposure to traditional media (television, radio, newspapers) and PCL-5 scores at the 6th month (*r* = .264, *p* = .037) and 9th month (*r* = .271, *p* = .032), as well as with treatment duration (*r* = .245, *p* = .050). Furthermore, there was no significant relationship between media type (traditional or social) and whether participants sought psychiatric treatment (χ² = 0.319, *p* = .375).

## 4 Discussion

A devastating natural disaster such as an earthquake can have psychological impacts that extend far beyond its epicenter. With the advancement of communication technologies, traumatic images and news can now be rapidly disseminated, reaching individuals in distant regions and entering virtually every home.

In our study, we aimed to explore how a major earthquake, occurring approximately a thousand kilometers away, affected individuals who had previously experienced a similar disaster within the same country. Specifically, longitudinal changes in post-traumatic symptom severity following secondary earthquake exposure and examined the contribution of clinical and sociodemographic factors to symptom trajectories. Several clinically meaningful findings emerged.

First, there was a robust overall decline in PTSD symptom severity over the 12-month follow-up, indicating partial natural recovery over time. This is consistent with longitudinal disaster psychiatry literature, which describes gradual symptom attenuation in a substantial proportion of disaster-exposed populations (19). However, the persistence of measurable symptoms in some subgroups highlights that recovery is neither uniform nor inevitable.

The Adult Resilience Scale (RSA) score showed the strongest independent association with PTSD symptom trajectories, explaining 40% of variance. This finding reinforces the growing body of evidence positioning psychological resilience as a key protective factor in trauma-exposed populations. Higher resilience may buffer individuals against the cumulative emotional load of repeated trauma exposure and facilitate adaptive coping. From a clinical perspective, this supports resilience-focused interventions as a potentially valuable component of post-disaster mental health care as suggested in literature (20).

Studies have shown that experiencing losses during an earthquake is associated with increased psychological distress in the aftermath (21). In particular, the loss of family members has been identified as a significant risk factor (22–25). Additionally, damage to or destruction of ones residence has also been shown to contribute substantially to post-disaster psychological problems (26–28). Consistent with these findings, our study revealed that individuals who reported experiencing such losses were significantly more likely to seek psychological treatment. Additionally, experiencing loss was also significantly associated with higher symptom severity. This aligns with the literature demonstrating that bereavement and material loss intensify trauma-related psychopathology. Loss may deepen the emotional meaning of subsequent disaster exposure, rendering individuals more vulnerable to re-experiencing, hyperarousal, and cognitive distortions (23). Clinically, this subgroup may benefit from more intensive monitoring and targeted grief-informed interventions following secondary disasters.

A central objective of this study was to examine the role of media exposure type on the post-traumatic symptom severity following secondary earthquake exposure. In a follow-up study with students in China reported that media exposure predicted possible PTSD symptoms months after the earthquake (29). Traditional media exposure was associated with significantly higher PTSD symptom severity than social media exposure, confirming media type as an independent psychosocial stressor beyond direct exposure.

Traditional media exposure was associated with significantly higher PTSD symptom severity than social media exposure, confirming media type as an independent psychosocial stressor beyond direct exposure. This finding contributes to a growing literature on the mental health consequences of indirect trauma. Research following the 2023 Kahramanmaraş earthquake suggested that repeated exposure to disaster-related media may exacerbate psychological distress, recommending media use be limited during such events to prevent future psychopathology (8). In our study, the main focus was to determine if the medium through which disaster-related information is consumed may function as an independent psychosocial stressor, influencing psychological burden beyond the effects of direct exposure alone. Importantly, individuals with different media exposure profiles showed differences in overall symptom severity, the course of symptom change over time was similar across groups, suggesting comparable recovery patterns regardless of the primary media source. This may indicate that although individuals exposed primarily to different types of media entered the follow-up period with differing symptom levels, there was no difference between the rate of symptom change over time, across groups. This pattern suggests that media exposure type may shape the initial intensity or activation of post-traumatic symptoms, rather than modifying the biological or psychological recovery processes that unfold over time. Traditional media and social media may differ in their capacity to amplify immediate distress without necessarily altering longer-term adaptation or habituation processes. Clinically, the differentiation between media types has important implications for assessment and intervention. Routine psychiatric evaluation following mass trauma events rarely includes a systematic assessment of media consumption patterns. The present findings suggest that such assessment may be beneficial. Psychoeducation regarding media consumption, strategies for limiting exposure, and structured guidance around adaptive information-seeking behaviors may represent simple yet underutilized components of trauma-informed care. Additionally, among participants who experienced loss during the Aegean Sea earthquake, exposure to traditional media showed modest correlations with higher PCL-5 scores at the 6th and 9th months and with longer treatment duration. These small effects do not imply causation; individuals with greater distress may simply have engaged more with media rather than media exposure exacerbating symptoms. Accordingly, these findings should be interpreted cautiously and considered preliminary.

Our findings suggest that receiving treatment after the initial earthquake associated with higher baseline post-traumatic symptom severity following secondary earthquake exposure, while simultaneously showing higher resilience scores and shorter subsequent treatment duration, highlights the complex and non-linear nature of trauma-related psychopathology. Rather than representing a simple risk or protective marker, prior treatment history appears to characterize a distinct clinical subgroup with both heightened vulnerability and enhanced adaptive resources. This aligns with previous research emphasizing the effectiveness of psychiatric treatment in alleviating post-disaster psychological symptoms (30–32). From a clinical psychopathology perspective, higher baseline symptom severity in previously treated individuals is consistent with models of stress sensitization and cumulative trauma burden, whereby repeated or unresolved traumatic experiences lower the threshold for symptom reactivation after subsequent stressors. Individuals with prior diagnoses or treatment may have neural and psychological systems that are more readily activated by trauma-related reminders, even when objective exposure is indirect. This is supported by literature demonstrating that previous episodes of PTSD or anxiety disorders increase the likelihood of more severe symptom re-emergence after re-exposure to trauma (33). At the same time, the finding of higher resilience scores in this group complicates a purely vulnerability-based interpretation. Higher resilience may reflect the acquisition of coping skills, cognitive frameworks, and emotional regulation strategies during earlier treatment processes (34). In this sense, resilience scores may be capturing not an absence of vulnerability, but a parallel development of psychological tools that coexist with underlying sensitivity. The shorter treatment duration observed after the second earthquake among individuals with a prior treatment history may be due to several reasons. One possible explanation may be, previously treated individuals may recognize symptom escalation earlier and seek help more promptly, resulting in more targeted and time-limited interventions. Also, familiarity with psychiatry or psychology clinics and healthcare workers may facilitate more efficient clinical engagement, reducing the need for prolonged treatment to achieve stabilization. The coexistence of higher resilience and higher symptom severity further underscores the need for multidimensional assessment approaches in post-disaster mental health care. Reliance on symptom severity alone may underestimate adaptive capacity, while exclusive focus on resilience may obscure persistent clinical risk. Future longitudinal studies should explicitly model this dual-domain structure, examining how vulnerability and resilience interact dynamically over time in trauma-exposed populations.

Previous studies have consistently found that women are at greater risk for experiencing psychological distress following an earthquake (6, 28, 35, 36). However, in our study, no significant gender differences were observed. This finding may be partly explained by the unequal gender distribution within our sample. Additionally, the geographical distance from the recent earthquakes epicenter and three year difference from the participants prior earthquake experience may have contributed to this result. Similarly, there is data in literature indicating that low education level is also a risk factor, our study did not find any results related to the duration of education (35).A recent meta-analysis on pharmacological treatments for PTSD reported that fluoxetine, paroxetine, sertraline, venlafaxine, and quetiapine yield small but positive effects in reducing symptom severity (37). Consistent with these findings, these medications were the most commonly prescribed in our study for participants requiring pharmacotherapy. In contrast, individuals with milder complaints, such as occasional insomnia or palpitations, were typically treated with symptomatic medications only. These participants demonstrated higher endurance scores and significantly lower PCL-5 scores throughout the study period. Notably, no increase in PTSD symptoms was observed in this subgroup during follow-up. These results support existing literature that suggests psychotropic medications should be reserved for cases with clear clinical indications, particularly in the early stages of post-trauma response, when overmedication may be unnecessary or even counterproductive (38). Although phenelzine was found to have better efficacy than other drugs in a network meta-analysis, it is not a widely preferred first-line drug as a routine treatment in Turkey (39). Therefore, pharmacotherapy may have been somewhat weak in terms of efficacy. However, studies have shown that psychotherapy is the gold standard in treatment. It is stated that the use of additional medication as pharmacotherapy can be helpful in the treatment of symptoms (40).

Our study has several limitations that should be considered when interpreting the findings. First, the sample size, although meeting the calculated requirement, was relatively small for detecting subtle associations, particularly in subgroup analyses, and was recruited solely from a psychiatry outpatient clinic, which may limit generalisability to the broader population. The sample was predominantly female, restricting the ability to examine gender-related differences. Second, data on media exposure, loss experiences, and PTSD symptoms were based on self-report, introducing potential recall and reporting biases. Not assessing baseline psychiatric comorbidity is also a limitation for interpretation. Another limitation is that individuals with relatives affected by the 2023 earthquake were excluded. While this decision helped isolate media-related effects by reducing additional trauma influences, it also restricts the generalizability of the findings, as the sample may not fully represent the broader population exposed to varying degrees of secondary impact. Finally, as an observational design, the study cannot establish a causal relationship between media exposure and PTSD symptom relapse.

## 5 Conclusion

Earthquakes exert profound psychological effects beyond their immediate physical destruction, reactivating PTSD symptoms through media-mediated secondary exposure in previously trauma-exposed individuals.This study demonstrates that traditional media exposure predicts higher PTSD symptom severity than social media, while prior psychiatric treatment identifies a high-risk yet resilient subgroup requiring shorter intervention duration post-retraumatization. Routine assessment of media exposure should be incorporated into post-disaster psychiatric evaluations, as media consumption can intensify distress and contribute to indirect trauma responses. In addition, proactively screening individuals with a history of trauma during subsequent disasters is essential, given their heightened vulnerability to cumulative stress. Resilience-focused interventions, may help buffer stress reactivity and strengthen adaptive coping capacities. Moreover, media-literacy based psychoeducation can mitigate the psychological impact of indirect trauma by supporting healthier patterns of media engagement. Taken together, these preliminary findings highlight the need for integrated public mental health strategies that combine early intervention, resilience training, and guidance on media consumption in disaster-prone regions. Future research should include multicenter trials featuring objective media-tracking methods and randomized early-intervention protocols to clarify causal mechanisms and optimize recovery trajectories.

## Data Availability

The raw data supporting the conclusions of this article will be made available by the authors, without undue reservation.
